# Construction of Recombinant *Mycobacterium smegmatis* Expressing the Pro-Apoptotic Protein BIK and Its Impact on Macrophage Apoptosis

**DOI:** 10.3390/pathogens15080857

**Published:** 2026-08-17

**Authors:** Yaning Pang, Jing Bi

**Affiliations:** National Clinical Research Center for Infectious Diseases, Guangdong Provincial Clinical Research Center for Tuberculosis, Shenzhen Third People’s Hospital, Southern University of Science and Technology, Shenzhen 518114, China; yaning2026@163.com

**Keywords:** tuberculosis, *Mycobacterium smegmatis*, ASP, BIK, apoptosis

## Abstract

Tuberculosis (TB), caused by *Mycobacterium tuberculosis*, remains a major global health threat. A key virulence mechanism involves the pathogen’s ability to inhibit apoptosis in infected macrophages, thereby facilitating immune evasion and persistent infection. The pro-apoptotic protein Bcl-2-interacting killer (BIK) represents a critical mediator of macrophage apoptosis. In this study, we engineered a recombinant *Mycobacterium smegmatis* strain (rMs-BIK) designed to secrete BIK via fusion with the Ag85B signal peptide (ASP). Secretion and expression of the BIK protein were confirmed by Western blotting. Quantitative flow cytometric analysis revealed that rMs-BIK induced significant apoptosis in RAW264.7 macrophages, with rates of 14.49%, 14.22%, and 22.24% at 12, 24, and 48 h post-infection, respectively. These values were markedly higher than those observed in cells infected with the wild-type strain (7.68%, 9.24%, and 10.90%) or the empty vector control (8.80%, 11.19%, and 14.43%). Our findings demonstrate the feasibility of functional pro-apoptotic protein delivery using a mycobacterial vector, providing a robust foundation for the future development of recombinant BCG-based vaccine candidates.

## 1. Introduction

Tuberculosis (TB), driven by *Mycobacterium tuberculosis* (*M. tuberculosis*), persists as a leading cause of morbidity and mortality worldwide. The clinical management of TB is further burdened by protracted treatment regimens, substantial healthcare costs, and a marked decline in patient quality of life [1,2,3]. Compounding this challenge, the emergence of drug-resistant *M. tuberculosis* strains and the syndemic of TB and HIV/AIDS have severely undermined current control strategies [4,5,6,7,8,9]. While Bacille Calmette-Guérin (BCG) remains the sole licensed vaccine against TB, its protective efficacy is predominantly confined to preventing severe pediatric forms of the disease; Critically, it fails to confer durable, reliable immunity in adults [10,11,12]. Consequently, the development of novel, effective, and safe TB vaccines has become a crucial scientific challenge that must be addressed as part of the global TB elimination efforts.

During *M. tuberculosis* infection, macrophages function as the primary cellular niche, serving a dual role as both the principal site for bacterial survival and replication, and as central effectors of the host immune defense. Induction of macrophage apoptosis is a critical host defense mechanism that not only eliminates intracellular pathogens but also potentiates antigen presentation—a prerequisite for mounting robust adaptive immune responses. Paradoxically, *M. tuberculosis* has exploited this vulnerability by evolving sophisticated immune evasion strategies over millennia, chief among them being the active suppression of host apoptosis to ensure chronic infection [13,14,15,16,17]. Notably, BCG, as an attenuated live vaccine, has partially retained this ability to inhibit apoptosis, which may contribute to its limited immunogenicity and variable protective efficacy in adults [18]. So finding ways to effectively induce macrophage apoptosis has become a promising strategy to make BCG more immunogenic.

Bcl-2-interacting killer (BIK) is a key pro-apoptotic member of the BH3-only subfamily in the Bcl-2 family, serving a vital regulatory function in the mitochondria-mediated intrinsic apoptotic pathway. BIK mostly facilitates apoptosis by specifically attaching to and inactivating anti-apoptotic members of the Bcl-2 family, such as Bcl-2 and Bcl-xL, through its BH3 domain. This connection efficiently alleviates the inhibition of the downstream pro-apoptotic effector molecules Bax and Bak, thereby facilitating mitochondrial outer membrane permeabilization and the release of cytochrome c, which then activates the caspase cascade and finally induces death [19,20,21,22]. Based on this potent pro-apoptotic capacity, we hypothesized that heterologous expression of BIK within BCG would overcome the vaccine’s inherent apoptosis-inhibitory phenotype. Such modulation is anticipated to potentiate antigen presentation and bolster protective immunity. To empirically test this hypothesis, we engineered a recombinant *Mycobacterium smegmatis* strain (rMs-BIK) designed to constitutively express and secrete functional BIK, and systematically evaluated its pro-apoptotic efficacy in murine RAW264.7 macrophages in vitro.

*Mycobacterium smegmatis* (*M. smegmatis*) shares substantial genetic, metabolic, and protein secretion system similarities with *M. tuberculosis,* but is distinguished by its rapid growth, safety in handling, and non-pathogenic nature. It has been widely used as a model organism in TB research and as a platform for vaccine development [23]. Notably, the Ag85B signal peptide (ASP) used in this study directs protein secretion via the Sec pathway, which is the primary protein export system in mycobacteria and has been demonstrated to be functionally active in BCG [24]. Therefore, the successful validation of ASP-mediated secretion in *M. smegmatis* provides a strong rationale for the transferability of this construct to BCG-based vaccine platforms. To enhance immune recognition of the target protein, the gene sequence encoding the signal peptide of the major *M. tuberculosis* secreted antigen Ag85b (ASP) was fused to the BIK gene, thereby generating a secretory BIK expression construct. This design enables the efficient secretion of the BIK protein from *M. smegmatis* via this signal peptide-directed pathway [25]. Subsequently, the ability of this recombinant strain to induce macrophage apoptosis was evaluated in vitro. In this study, we aimed to test whether a functional pro-apoptotic protein can be successfully expressed and secreted from a mycobacterial vector, as a first-step validation toward the eventual evaluation of this approach in BCG-based vaccine platforms.

## 2. Materials and Methods

### 2.1. Bacterial Strains, Cells, and Plasmids

*Mycobacterium smegmatis* mc^2^ 155 (hereafter *M. smegmatis*), *Escherichia coli* DH5α competent cells, and the murine macrophage cell line RAW264.7 were procured from the American Type Culture Collection (ATCC) or obtained from established institutional stock centers and routinely maintained in the laboratory. The *E. coli*-mycobacterial shuttle plasmid pMV261-10His was preserved in our laboratory. The reference strain *M. tuberculosis* H37Rv was acquired from the institutional strain repository (Shenzhen Third People’s Hospital, Shenzhen, China).

### 2.2. Experimental Animals

Female BALB/c mice (6-8 weeks old), specific pathogen-free (SPF), were obtained from the institutional experimental animal center. The animal study protocol was approved by the Institutional Animal Care and Use Committee of Shenzhen Third People’s Hospital.

### 2.3. Molecular Cloning and Nucleic Acid Extraction Reagents

Phusion High-Fidelity DNA Polymerase (Thermo Fisher Scientific, Waltham, MA, USA); Plasmid Miniprep Kit, Universal DNA Purification Kit, TRNzol Universal Total RNA Extraction Reagent, (Tiangen Biotech, Beijing, China); DNA Marker, ONE-Step gDNA Removal and cDNA Synthesis SuperMix, 6×Loading Buffer (TransGen Biotech, Beijing, China); Gibson Assembly^®^ Master Mix (New England Biolabs, Ipswich, MA, USA); Mouse Lymphocyte Separation Medium (Dakewe Biotech, Shenzhen, China).

### 2.4. Protein Analysis Reagents

5× Protein Loading Buffer, Skimmed milk powder, Bovine serum albumin (BSA), Tryptone, Yeast extract, Sodium chloride, Agar, Acrylamide, N,N’-Methylenebisacrylamide, Ammonium persulfate, Tetramethylethylenediamine (TEMED) (Solarbio, Beijing, China); BCA Protein Assay Kit, Prestained Protein Ladder, Sodium dodecyl sulfate (SDS), Glycine (Thermo Fisher Scientific, Waltham, MA, USA); ECL Chemiluminescence Substrate (Sangon Biotech, Shanghai, China); Anti-His Tag Mouse Monoclonal Antibody (Beyotime Biotechnology, Shanghai, China); Horseradish peroxidase (HRP)-conjugated Goat Anti-Mouse IgG Secondary Antibody (Servicebio, Wuhan, China); Methanol, Glacial acetic acid, Tween-20 (Sinopharm Chemical Reagent, Shanghai, China).

### 2.5. Culture Media and Antibiotics

Middlebrook 7H9 Broth, Middlebrook 7H10 Agar, ADC Enrichment (BD Biosciences, Franklin Lakes, NJ, USA); DMEM (high glucose) medium, Premium-grade Fetal Bovine Serum (FBS), Penicillin-Streptomycin (P/S) solution (Thermo Fisher Scientific, Waltham, MA, USA); Kanamycin, Gentamicin (Solarbio, Beijing, China); Tween-80, Glycerol (Sangon Biotech, Shanghai, China).

### 2.6. Buffers and Chemical Reagents

Phosphate-Buffered Saline (PBS), Protease Inhibitor Cocktail (Solarbio, Beijing, China); Tris base, Ethylenediaminetetraacetic acid (EDTA), Sodium carbonate, Sodium hydroxide, Sodium bicarbonate (Sangon Biotech, Shanghai, China); Annexin V-FITC/7-AAD Apoptosis Detection Kit (BD Biosciences, Franklin Lakes, NJ, USA); Lipopolysaccharide (LPS) (Sigma-Aldrich, St. Louis, MO, USA).

### 2.7. Instruments

Flow cytometer (BD FACSCanto™ II, BD Biosciences, Franklin Lakes, NJ, USA); Chemiluminescence imaging system (WD-9413B, Beijing Liuyi Biotechnology, Beijing, China); Thermal cycler (S1000, Bio-Rad, Hercules, CA, USA); Gel documentation system (WD-9413B, Beijing Liuyi Biotechnology, Beijing, China); Microvolume spectrophotometer (NanoDrop2000, Thermo Fisher Scientific, Waltham, MA, USA); High-speed refrigerated centrifuge (D-37520, Sigma, Osterode, Germany); Incubator shaker (HYG-C3, Taicang Haocheng, Taicang, China); CO_2_ incubator (GSP-70, Tianjin Laibotep, Tianjin, China); Ultrasonic cell disruptor (SCIENTZ-IID, Scientz, Ningbo, China); Electroporator (Gene Pulser^®^ Xcell™, Bio-Rad, Hercules, CA, USA), Vertical electrophoresis and transfer system (DYY-6C, Beijing Liuyi Biotechnology, Beijing, China); Ultrafiltration centrifugal tubes (10 kDa molecular weight cut-off; Millipore, Burlington, MA, USA); Bacterial sonication and counting system (Ningbo Scientz Biotechnology Co., Ltd., Ningbo, China).

### 2.8. Culture of M. tuberculosis and Genomic DNA Extraction

The standard strain H37Rv of *M. tuberculosis* was first cultured in Middlebrook 7H9 liquid medium supplemented with 10% Middlebrook ADC enrichment broth. The culture was incubated in a constant-temperature shaking incubator at 37 °C and 80 r/min for three weeks. Once it reached the mid-logarithmic growth phase, the culture was inactivated by heating at 80 °C for 2–3 h. Genomic DNA was subsequently isolated using a thermal lysis protocol. Briefly, 50 mL of the inactivated culture was centrifuged at 12,000× *g* for 10 min at 4 °C, and the supernatant was discarded. The pelleted bacteria were thoroughly resuspended in 2.5 mL of sterile double-distilled water and then boiled in a 100 °C water bath for 10 min. This was followed by centrifugation at 12,000× *g* for another 10 min. The supernatant containing the crude genomic DNA extract was collected, aliquoted, and stored at −20 °C for later use. All experiments with viable *M. tuberculosis* H37Rv were performed in a certified Biosafety Level 3 (BSL-3) facility in compliance with institutional and national biosafety regulations.

### 2.9. Synthesis of cDNA from Mouse Spleen Lymphocytes

Total RNA was extracted from murine splenocytes following a standardized protocol. Briefly, spleens were aseptically harvested from euthanized female BALB/c mice and mechanically dissociated through a 200-mesh nylon cell strainer using the plunger of a 5 mL syringe in the presence of lymphocyte separation medium. Mononuclear cells were then enriched by density gradient centrifugation over RPMI-1640 medium at 800× *g* for 30 min at room temperature. Following collection of the interphase lymphocyte layer and a washing step, total RNA was isolated using TRNzol Universal Reagent according to the manufacturer’s instructions. Specifically, cell pellets were lysed in 1 mL TRNzol, followed by phase separation upon addition of 200 µL chloroform and vigorous vortexing. After incubation at room temperature for 3 min, samples were centrifuged at 12,000× *g* for 15 min at 4 °C. The aqueous phase was collected, and RNA was precipitated with an equal volume of isopropanol at −20 °C for 30 min. The resulting RNA pellet was washed once with 75% (*v*/*v*) ethanol, air-dried, and resuspended in 30 µL of RNase-free water. RNA concentration and purity were assessed using a microvolume spectrophotometer, with acceptable quality defined by an A260/A280 ratio between 1.8 and 2.1. Subsequently, first-strand cDNA was synthesized from 6 µL of total RNA using the One-Step gDNA Removal and cDNA Synthesis SuperMix kit (TransGen Biotech, Beijing, China). The 20 µL reaction system comprised 1 µL Anchored Oligo(dT_18_) Primer, 10 µL 2× TS Reaction Mix, 1 µL TransScript^®^ RT/RI Enzyme Mix, 1 µL gDNA Remover, and nuclease-free water. Thermal cycling conditions included an initial denaturation at 65 °C for 5 min, reverse transcription at 42 °C for 30 min, and enzyme inactivation at 85 °C for 5 s. The synthesized cDNA was aliquoted and stored at −20 °C pending downstream applications.

### 2.10. Construction of Recombinant Plasmid pMV261-ASP-BIK-10His

To construct the fusion expression plasmid, primer pairs targeting the ASP and BIK genes were designed and synthesized. The primer sequences were as follows:

ASP gene:

Forward: 5′-CCAAGACAATTGCGGATATGACAGACGTGAGCCGAAAGAT-3′
Reverse: 5′-TGGAAAGCTTCGAATTCCGCGCCCGCGGTTGCCGCTC-3′

BIK gene:

Forward: 5′-CCGCGGGCGCGGAATTCATGTCGGAGGCGAGACTTATGGC-3′
Reverse: 5′-TGGTGGTGGAAAGCTTCCTGAAGCTGCAAATACCAGGCCC-3’

The target gene fragments for ASP and BIK were amplified by PCR using H37Rv genomic DNA and mouse spleen lymphocyte cDNA as templates, respectively. After purification, the two gene fragments were directionally assembled with the linearized pMV261-10His vector plasmid using a seamless cloning kit to construct the recombinant plasmid pMV261-ASP-BIK-10His. The recombinant product was transformed into competent *E. coli* cells and spread onto LB plates containing the corresponding antibiotic. Plates were incubated overnight at 37 °C. A single colony was picked and inoculated into liquid LB medium, which was then shaken at 37 °C and 200 r/min until it reached the logarithmic growth phase. The plasmid was extracted from the bacterial culture using a plasmid mini-prep kit. Plasmid size was preliminarily verified by agarose gel electrophoresis. The plasmid was sequenced using universal pMV261-10His vector primers (forward: 5′-ATGGCCAAGACAATTGCGGATATGA-3′; reverse: 5′-TTAGTGATGGTGGTGATGGTGGTGA-3′) to confirm the correct insertion of the ASP and BIK genes and the integrity of the reading frames, thereby validating the successful construction of the recombinant plasmid.

### 2.11. Preparation of M. smegmatis Competent Cells

A fresh single colony of *M. smegmatis* mc^2^155 was inoculated into 5 mL of 7H9 liquid medium and cultured at 37 °C with shaking at 200 r/min until the logarithmic growth phase (OD_600_ ≈ 0.5–1.0). For scale-up, the culture was subcultured at a 1:100 ratio into 100 mL of fresh 7H9 medium and shaken overnight under the same conditions until the OD_600_ reached approximately 0.6. The bacterial suspension was pre-chilled on ice for 30–60 min and then centrifuged at 4 °C and 5000× *g* for 10 min to collect the cell pellet. The resulting pellet was resuspended in ice-cold 10% sterile glycerol and washed three times to thoroughly remove medium components. Following the final wash, cells were resuspended in ice-cold 10% glycerol, aliquoted into sterile 1.5 mL centrifuge tubes (200 µL per tube), and stored at −80 °C for future use.

### 2.12. Electrotransformation of Recombinant Plasmids

Electrotransformation of *M. smegmatis* mc^2^155 competent cells was performed using both the sequence-verified recombinant plasmid pMV261-ASP-BIK-10His and the empty vector control pMV261-10His. A suitable amount of plasmid (approximately 60 ng/μL) was mixed with 200 μL of competent cells on ice and transferred into a pre-chilled 2 mm electroporation cuvette. Electroporation was performed using an electroporator set at 2.5 kV, 1000 Ω resistance, and 25 μF capacitance. Immediately after electroporation, 1 mL of pre-warmed 7H9 medium was added, gently mixed, and the mixture was incubated at 37 °C for 2–3 h for recovery. The recovered culture was evenly spread onto 7H10 solid agar plates containing a final concentration of 20 µg/mL kanamycin and incubated in a 37 °C incubator for 3 to 5 days until distinct single colonies were clearly visible.

### 2.13. Selection and Identification of Successful M. smegmatis Transformants

Putative transformants were selected from 7H10 agar plates containing 20 µg/mL kanamycin and inoculated into 5 mL of liquid 7H9 medium supplemented with the same antibiotic concentration. Cultures were incubated at 37 °C with shaking at 200 r/min for 2–3 days. An appropriate amount of bacterial cells was collected, boiled, and the supernatant was used as a template. PCR amplification was performed using universal primers for the pMV261-10His vector. Specific bands of the expected size were detected by agarose gel electrophoresis, providing preliminary confirmation that the recombinant plasmid or empty vector had been successfully introduced into *M. smegmatis*. Subsequently, PCR-positive products were submitted for sequencing. Comparison with the target sequences ultimately confirmed the correct integration and sequence integrity of the ASP and BIK genes in the recombinant strain.

### 2.14. Collection of Cytoplasmic and Culture Medium Protein Samples

Recombinant *M. smegmatis* strains harboring plasmids pMV261-ASP-BIK-10His and pMV261-10His, along with the wild-type strain (control), were cultured as follows: Recombinant strains were inoculated into 100 mL of 7H9 liquid medium containing 20 µg/mL kanamycin, whereas the wild-type strain was inoculated into an equal volume of antibiotic-free medium. All cultures were incubated at 37 °C and 200 r/min until the OD600 reached 0.6–0.8. Fifty milliliters of culture from each group was centrifuged at 4 °C and 4000× *g* for 15 min to separate the supernatant (culture medium sample) from the bacterial pellets. The pellets were resuspended in 30 mL of pre-chilled sterile PBS and sonicated on ice (5 s pulse on, 10 s pulse off, for a total duration of 30 min). Subsequently, the lysate was centrifuged at 4 °C and 10,000× *g* for 15 min. The collected supernatant served as the cytoplasmic protein sample. Both the culture supernatants and cytosolic fractions were supplemented with 1× protease inhibitor cocktail, filter-sterilized through a 0.22 µm filter, and concentrated using 10 kDa molecular weight cut-off ultrafiltration centrifugal tubes at 4 °C and 4000× *g*. The concentrated protein samples were kept on ice for immediate use or stored at −20 °C.

### 2.15. Detection of Protein Expression Levels

Protein concentrations of the concentrated samples were determined using the BCA assay. An equal amount of protein (5 µg per lane) was loaded for subsequent analysis. Proteins were separated by SDS-polyacrylamide gel electrophoresis (SDS-PAGE) using gels composed of a 5% stacking gel and a 15% separating gel. Electrophoresis was performed initially at 80 V through the stacking gel and then at 120 V through the separating gel until the bromophenol blue dye front reached the bottom of the gel. Following electrophoresis, proteins were transferred onto a PVDF membrane using a wet transfer system at a constant current of 250 mA for 30 min. The membrane was blocked with 5% (*w*/*v*) skim milk in TBST (Tris-buffered saline containing 0.05% Tween 20) for 2 h at room temperature. The membrane was then incubated overnight at 4 °C with an anti-His tag mouse monoclonal primary antibody diluted 1:1500 in the blocking buffer. After washing four times with TBST (10 min per wash), the membrane was incubated with an HRP-conjugated goat anti-mouse IgG secondary antibody diluted 1:5000 for 1 h at room temperature. After additional washes with TBST, the membrane was incubated with a chemiluminescent substrate (e.g., ECL), and signals were detected using a chemiluminescence imaging system. The expression levels of the target protein were quantified using image analysis software. Western blot images were minimally adjusted for brightness/contrast using ImageJ 1.53e (National Institutes of Health, Bethesda, MD, USA) with identical settings applied to the whole blot; no local retouching was performed.

### 2.16. Infection of RAW264.7 Macrophages with Recombinant M. smegmatis and Apoptosis Detection

#### 2.16.1. Cell Culture

RAW264.7 mouse macrophages were routinely maintained in DMEM medium supplemented with 10% fetal bovine serum (FBS) and 1% penicillin-streptomycin (100 U/mL penicillin and 100 µg/mL streptomycin) at 37 °C in a humidified atmosphere containing 5% CO_2_. Cells were passaged to achieve the desired confluence prior to experiments. Logarithmic-phase cells were harvested, resuspended, and adjusted to a density of 1.5 × 10^6^ cells/mL. Lipopolysaccharide (LPS) was added to a final concentration of 1 μg/mL. The cell suspension was then seeded uniformly into six-well plates at 2 mL per well (equivalent to 3 × 10^6^ cells per well) and stimulated with LPS for 24 h. Subsequently, the medium was replaced with antibiotic-free complete DMEM, and cells were cultured for an additional 24 h before infection.

#### 2.16.2. Bacterial Preparation and Infection

Wild-type *M. smegmatis* mc^2^155, the empty vector control strain (pMV261-10His), and the recombinant strain (pMV261-ASP-BIK-10His) were cultured to mid-logarithmic phase (OD_600_ ≈ 1.0). Bacterial cells were washed with antibiotic-free DMEM by centrifugation at 13,000 r/min for 10 min to remove residual 7H9 broth components. The pellets were resuspended in 2 mL of antibiotic-free complete DMEM, transferred to a sonication tube, and subjected to sonication using a bacterial sonication and counting system (Ningbo Scientz Biotechnology Co., Ltd., China) to disperse bacterial clumps. The system is equipped with a sonicator probe and a counting software module that estimates bacterial concentration based on the correlation between OD600 and colony-forming units (CFUs). Bacterial concentration was determined by measuring OD600 or by plating for colony-forming units (CFUs). RAW264.7 cells were infected with bacterial strains at a multiplicity of infection (MOI) of 10, corresponding to a bacteria-to-cell ratio of 10:1. Briefly, 3 × 10^7^ bacteria (quantified using a bacterial sonication and counting system) were added to 2 mL of antibiotic-free DMEM complete medium and mixed thoroughly. The culture supernatant was removed from the six-well plates containing pre-plated cells, and the bacterial suspension (2 mL per well) was added. After incubation at 37 °C for 2 h, the supernatant was discarded. The cells were then washed three times with PBS, and the medium was replaced with DMEM complete medium containing 50 μg/mL gentamicin. The cells were further cultured at 37 °C for 12, 24, 48, and 72 h. Quantitative data for apoptosis rates are presented as medians with interquartile ranges (IQRs). Group comparisons were performed using the Kruskal–Wallis test followed by Dunn’s post hoc test (for multiple group comparisons). Statistical analyses were conducted using GraphPad Prism (version 9.0) (GraphPad Software, San Diego, CA, USA). Differences were considered statistically significant at *p* < 0.05.

#### 2.16.3. Detection of Apoptosis by Annexin V-FITC/7-AAD Double Staining

At the indicated time points, cells were collected by discarding the culture supernatant and washing once with PBS to remove residual serum. Subsequently, 1 mL of trypsin was added, and the cells were incubated at 37 °C for 1 min. Digestion was terminated by adding 3 mL of DMEM complete medium. The detached cells were gently resuspended and transferred to a 15 mL centrifuge tube, followed by centrifugation at 500× *g* for 5 min. The supernatant was removed, and the cell pellet was resuspended in 1 mL of ice-cold PBS (4 °C) and transferred to an EP tube. The cells were then centrifuged at 300× *g* for 3 min, washed once more with PBS, and centrifuged again at 300× *g* for 3 min. The PBS supernatant was discarded, and the cells were resuspended in 100 μL of 1× binding buffer and transferred to flow cytometry tubes. For dilution, 20 μL of the cell suspension was transferred to a new flow cytometry tube and mixed with 80 μL of 1× binding buffer. From this mixture, 20 μL was further transferred to another tube and mixed with 80 μL of 1× binding buffer, resulting in a 25-fold dilution.

The following control groups were included: blank control (no staining), Annexin V-FITC single-stained control, and 7-AAD single-stained control. For experimental samples, cells were stained with both Annexin V-FITC and 7-AAD, incubated at room temperature in the dark for 10 min, and then immediately analyzed by flow cytometry using a BD FACSCanto™ II flow cytometer (BD Biosciences, San Jose, CA, USA).

## 3. Results

### 3.1. Amplification of ASP and BIK Gene Fragments

**Figure 1:**
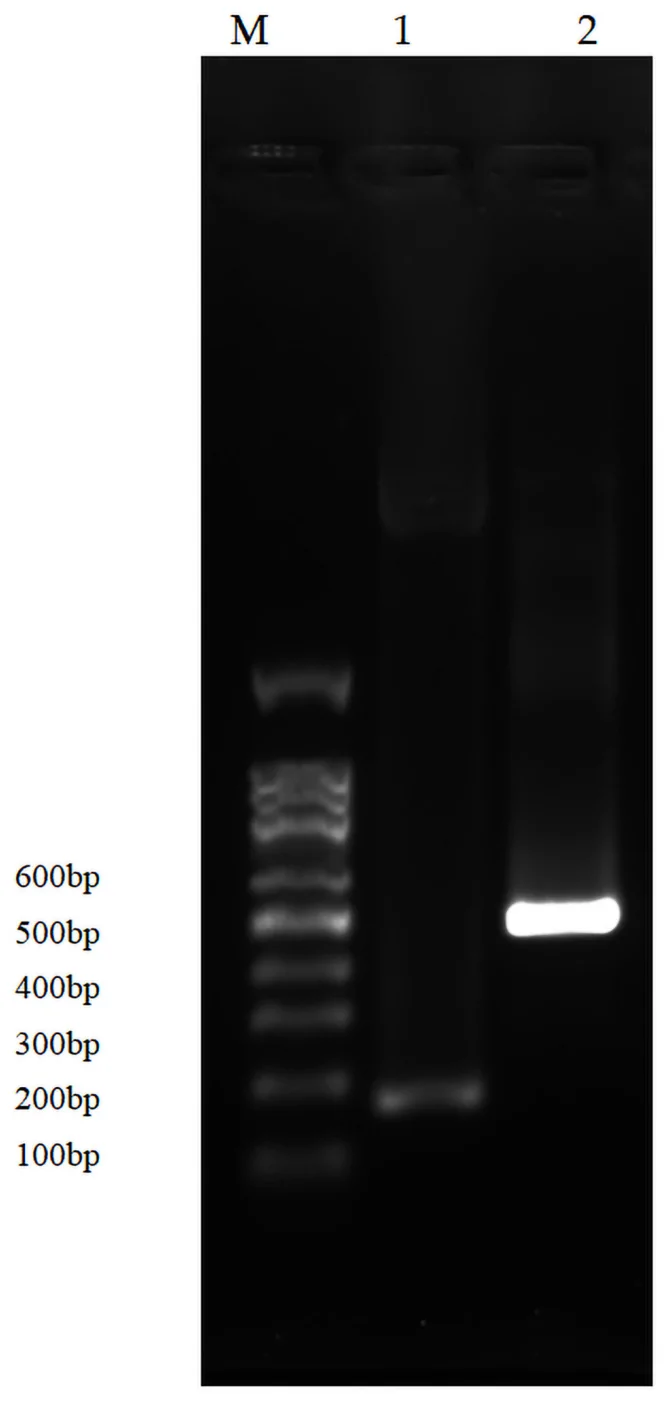
PCR amplification of ASP and BIK gene fragments. Legend: M, DNA molecular weight marker; Lane 1, ASP fragment (156 bp); Lane 2, BIK fragment (484 bp). The gel shows single bands of expected sizes without non-specific amplification. The two bands were resolved in separate wells of the same 1.5% agarose gel.

The ASP and BIK gene fragments were successfully amplified by PCR using genomic DNA from *M. tuberculosis* H37Rv and cDNA from murine splenocytes as templates, respectively. For the signal peptide-encoding sequence, a 156 bp ASP fragment was amplified using the H37Rv genome as the template. For the pro-apoptotic protein-coding sequence, a 484 bp BIK fragment was amplified using mouse cDNA as the template. Agarose gel electrophoresis results (Figure 1) showed sharp, single bands without non-specific amplification, confirming the successful and specific amplification of both ASP and BIK gene fragments for subsequent recombinant plasmid construction.

### 3.2. Construction of the Recombinant Plasmid and Its Transformation into Mycobacterium smegmatis

**Figure 2:**
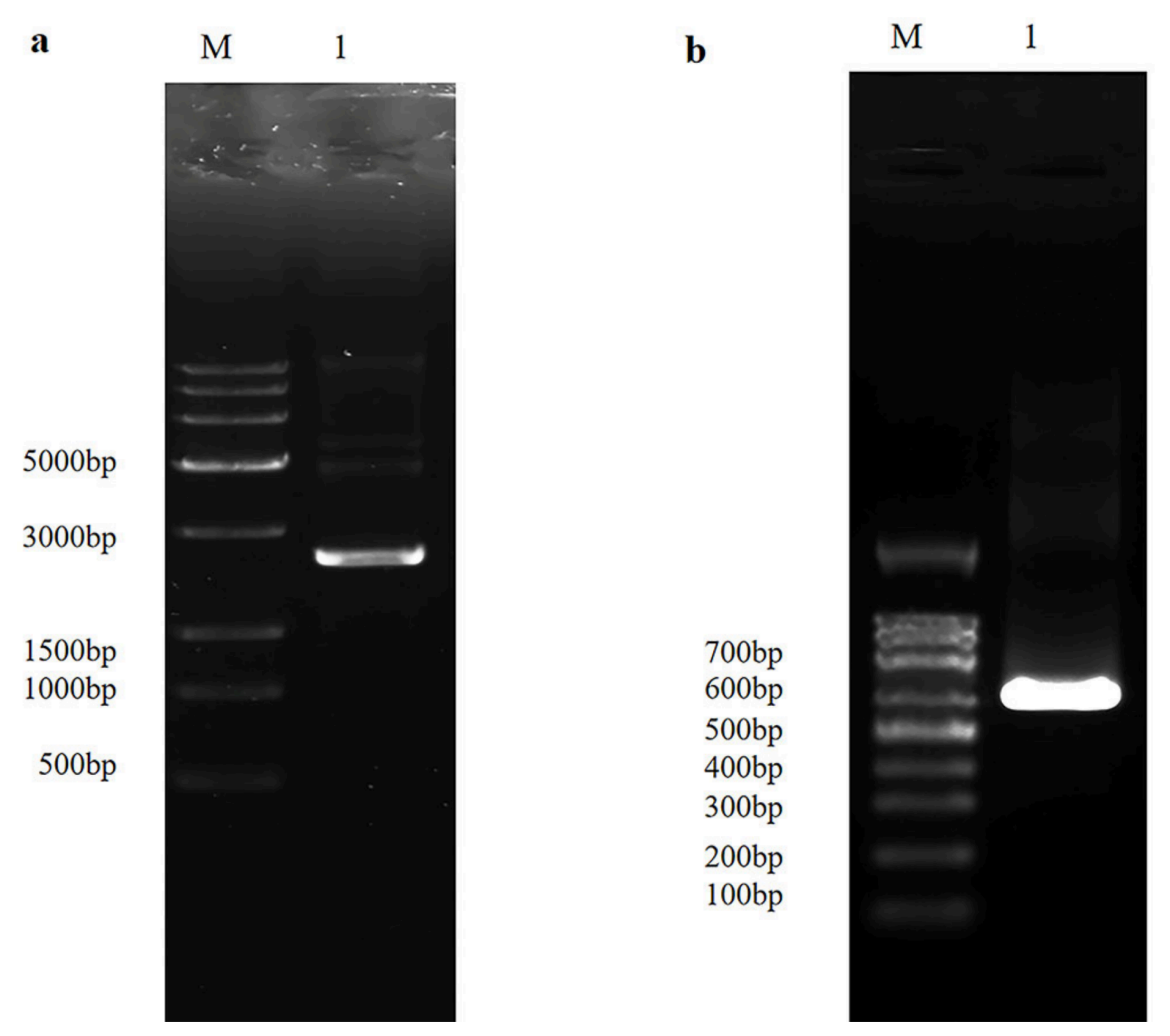
(**a**) Agarose gel electrophoresis of the recombinant plasmid pMV261-ASP-BIK-10His extracted from *E. coli*. M: DNA marker; Lane 1: pMV261-ASP-BIK-10His (~5.1 kb, supercoiled form). (**b**) PCR verification of the recombinant plasmid in *M. smegmatis transformants*. M: DNA marker; Lane 1: PCR-amplified fragment (639 bp). Cropped panels are derived from the same original gel.

The recombinant expression plasmid pMV261-ASP-BIK-10His was engineered by seamlessly cloning the ASP and BIK gene fragments into the pMV261-10His backbone. Following propagation in *E. coli*, plasmid extraction and restriction analysis revealed a distinct ~5.1 kb band by agarose gel electrophoresis, consistent with the predicted size of the construct (Figure 2a), thereby confirming successful assembly. To screen for successful transformants following electroporation into M. smegmatis mc^2^155, bacterial colonies were subjected to PCR analysis using primers specific to the pMV261 backbone. This screening yielded a specific ~639 bp amplicon corresponding to the ASP-BIK fusion junction (Figure 2b), indicating that the recombinant plasmid had been successfully introduced into *M. smegmatis*.

### 3.3. Expression and Secretion of the Recombinant BIK Protein

**Figure 3:**
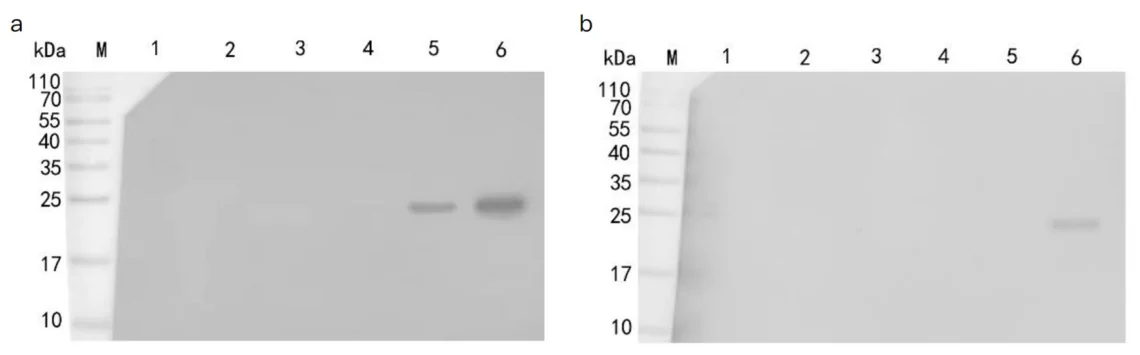
Western blot analysis of BIK protein expression and secretion. Legend: (**a**) Cytoplasmic fractions: M: protein molecular weight marker; Lane 1-2, wild-type *M. smegmatis*; Lane 3-4, empty vector control (pMV261-10His); Lanes 5-6, recombinant strain (pMV261-ASP-BIK-10His). (**b**) Culture supernatant fractions: M: protein molecular weight marker; Lane 2, wild-type; Lane 4, empty vector; Lane 6, recombinant strain. The His-tagged BIK protein (~25 kDa) is detected only in recombinant strain samples, confirming successful expression and ASP-mediated secretion.

Western blot analysis (Figure 3a,b) revealed that the target protein band was detected specifically only in *M. smegmatis* samples harboring the pMV261-ASP-BIK-10His recombinant plasmid. This protein band was present in both the bacterial lysate and culture supernatant of the recombinant strain, with an apparent molecular weight as expected. In contrast, no corresponding band was detected in samples from the wild-type strain or the control strain transformed with the empty vector (pMV261-10His). This result indicates that the ASP signal peptide-BIK fusion protein was successfully expressed in recombinant *M. smegmatis*. More importantly, the BIK protein was detected in the extracellular medium, confirming that the engineered ASP effectively mediated the transmembrane transport and secretion of BIK. This demonstrates the successful extracellular secretion of the pro-apoptotic protein BIK by recombinant *M. smegmatis*.

### 3.4. Quantitative Analysis of Macrophage Apoptosis

**Figure 4:**
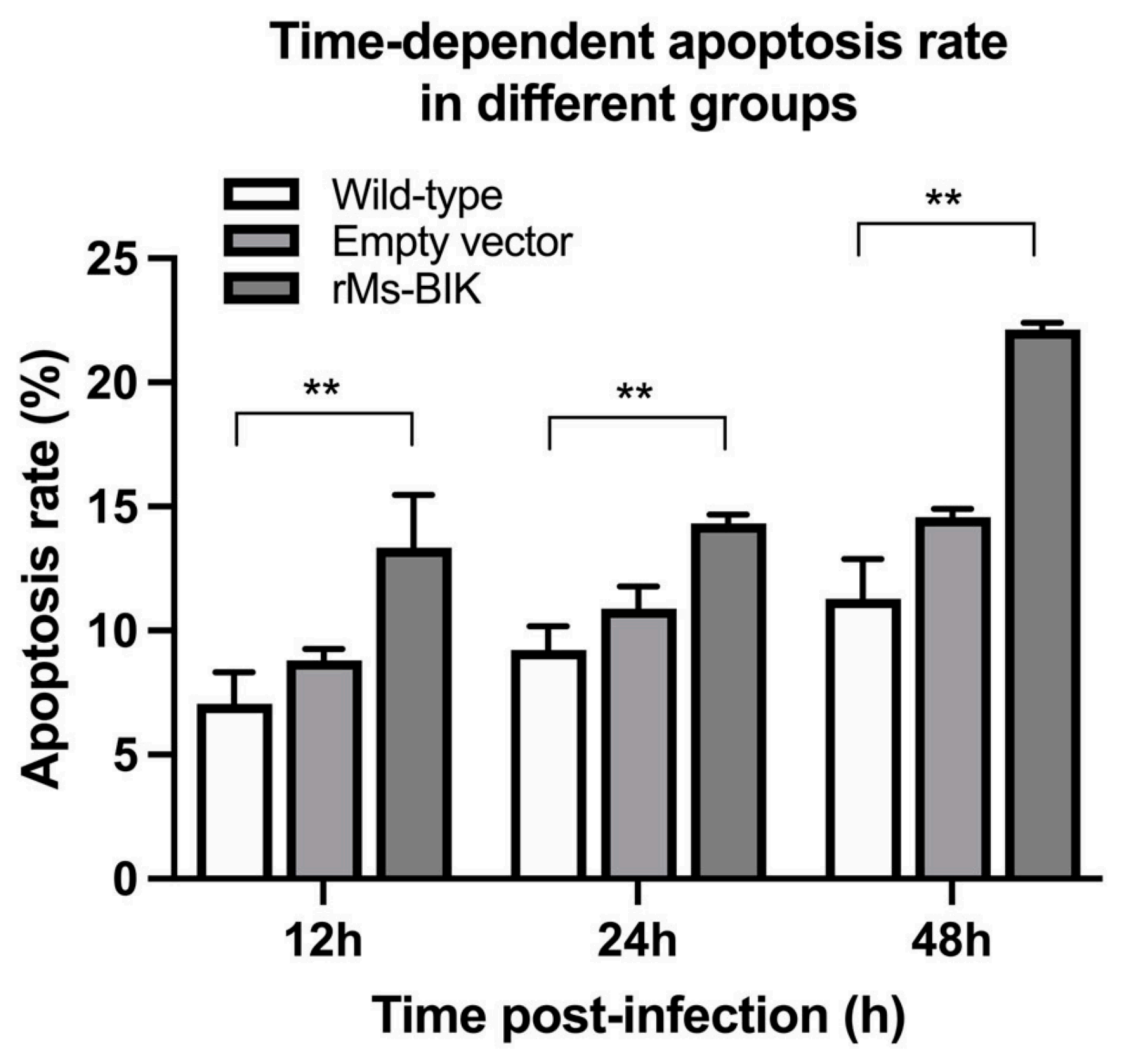
Quantitative comparison of apoptosis rates induced by different *M. smegmatis* strains. Legend: RAW264.7 cells were infected with WT, EV, or rMs-BIK strains for 2 h, followed by flow cytometric analysis at 12, 24, and 48 h. Data are presented as median values with interquartile ranges (IQR) from three independent biological replicates. Statistical significance was determined by the Kruskal–Wallis test followed by Dunn’s post-hoc test; Exact *p* values are provided in the text. ** *p* < 0.01.

**Figure 5:**
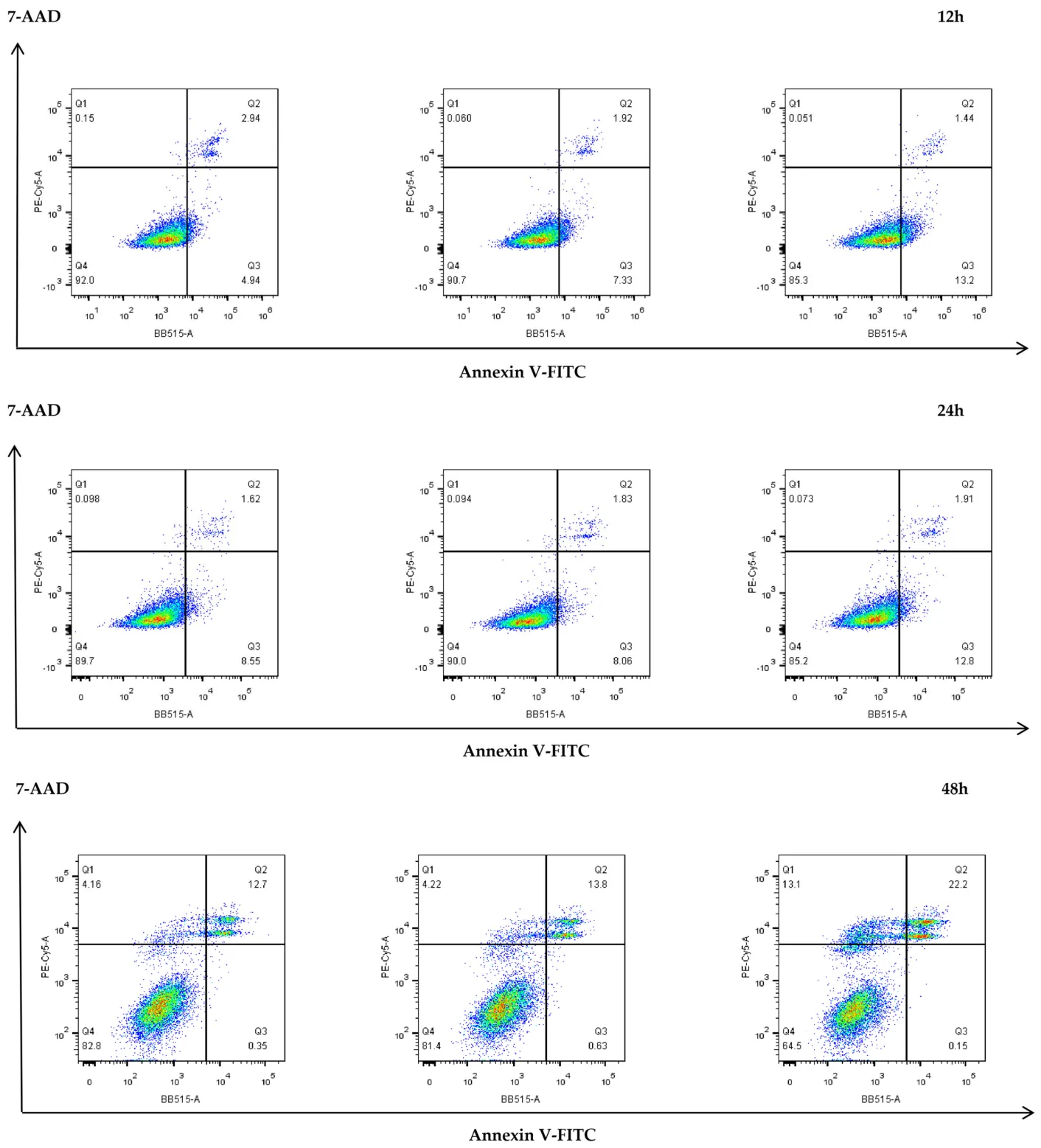
Representative flow cytometry plots of macrophage apoptosis. Legend: For each time point (12 h, 24 h, and 48 h), the plots are arranged from left to right as follows: wild-type *M. smegmatis*, empty vector control (pMV261−10His), and recombinant strain (rMs−BIK, pMV261−ASP−BIK−10His). In each plot, the four quadrants are defined as follows: Q1 (upper left), necrotic cells; Q2 (upper right), late apoptotic cells; Q3 (lower right), early apoptotic cells; Q4 (lower left), live cells. Representative plots from *n* = 3 independent infections.

To evaluate the pro-apoptotic efficacy of the ASP-BIK recombinant strain, RAW264.7 macrophages were infected with various *M. smegmatis* strains at a multiplicity of infection (MOI) of 10 for 2 h. Following the removal of extracellular bacteria with gentamicin-containing medium, cells were harvested at 12, 24, and 48 h post-infection and analyzed for apoptosis by flow cytometry using Annexin V-FITC and 7-AAD staining. Quantitative analysis revealed a distinct temporal increase in apoptosis rates across all groups. Notably, infection with the rMs-BIK strain resulted in significantly elevated apoptosis compared to both the wild-type (WT) and empty vector (EV) controls at all tested time points (*p* < 0.05 at 12 h and 24 h; *p* < 0.001 at 48 h). Specifically, median apoptosis rates in the rMs-BIK group reached 14.49% (IQR: 10.87–14.64%), 14.22% (IQR: 14.03–14.71%), and 22.24% (IQR: 21.82–22.35%) at 12, 24, and 48 h, respectively, whereas corresponding values for the WT and EV groups ranged from 7.68% to 10.90% and 8.80% to 14.43%, respectively. To visually emphasize the comparative efficacy, the data are presented as median values with interquartile ranges in Figure 4. Representative raw cytometry plots are provided in Figure 5. Collectively, these findings demonstrate that heterologous expression of the ASP-BIK fusion protein robustly potentiates macrophage apoptosis, validating the functional delivery of BIK via the recombinant mycobacterial vector.

## 4. Discussion

A cardinal virulence strategy of *M. tuberculosis* involves the active suppression of macrophage apoptosis to evade immune surveillance and establish chronic infection [26,27]. The restricted protective effectiveness of BCG in adults is partially ascribed to its sustained capacity to inhibit host cell apoptosis. BIK, a crucial pro-apoptotic factor, selectively binds to and neutralizes anti-apoptotic proteins, including BCL-2 and BCL-XL, thereby lessening their suppression of the mitochondrial apoptotic pathway and ultimately triggering cellular apoptosis [28,29]. It is well established in the literature that macrophage apoptosis can enhance antigen presentation and facilitate adaptive immune responses. However, the present study was designed to address a more fundamental question: whether a mycobacterial vector can deliver a functional pro-apoptotic protein to macrophages. Our data provide a definitive affirmative response to this question, establishing the feasibility of functional protein transduction via this recombinant platform.

In this study, we engineered a recombinant *M. smegmatis* strain (rMs-BIK) capable of secreting the BIK fusion protein. Western blot analysis confirmed that BIK was successfully expressed and secreted into the culture supernatant. Flow cytometry analysis demonstrated that rMs-BIK elicited substantially higher apoptotic rates in RAW264.7 macrophages compared to the wild-type strain and empty vector control at 12, 24, and 48 h. This confirms that the secreted BIK retains its biological activity after translocation across the mycobacterial cell wall, indicating that the ASP-BIK fusion strategy does not interfere with proper protein folding or function. Importantly, this finding extends the utility of *mycobacterial* vectors beyond the delivery of bacterial antigens, demonstrating that they can serve as vehicles for delivering functional mammalian pro-apoptotic proteins directly to macrophages—the primary host cells of *M. tuberculosis* infection. This vector-based delivery strategy ensures that the pro-apoptotic effect is confined to phagocytic cells, providing a built-in cell-type specificity that would be difficult to achieve with conventional protein delivery methods.

*M. smegmatis* exhibits significant genetic, metabolic, and protein secretion system similarities with BCG [23], while providing rapid growth, safe handling, and non-pathogenic characteristics. Notably, the Ag85B signal peptide (ASP) used in this study directs protein secretion via the Sec pathway, which is the primary protein export system in *mycobacteria* and has been demonstrated to be functionally active in BCG [24]. Thus, the successful secretion of BIK using ASP in *M. smegmatis* strongly suggests that the same construct would be recognized and processed by the Sec machinery in BCG. This renders *M. smegmatis* an effective surrogate vector for the preliminary validation of the ASP-BIK expression and secretion system. *Mycobacteria* are preferentially absorbed by phagocytic cells, hence predominantly targeting macrophages, the principal host cells of *M. tuberculosis*. Monocytes and dendritic cells may also internalize the vaccination strain, while non-phagocytic cells remain unaffected [30,31]. Recent advances in mycobacterial vaccine development have leveraged pro-apoptotic strategies to potentiate immunogenicity. The recombinant BCG strain VPM1002 (rBCGΔureC::hly) promotes antigen cross-presentation by disrupting the phagosomal membrane via listeriolysin O, thereby facilitating CD8^+^ T cell responses [32]. Our study differs from this approach in a key respect: VPM1002 relies on physical disruption of the phagosome to release bacterial antigens into the cytosol, whereas we employed BIK, a BH3-only protein that acts on the host mitochondrial apoptotic pathway by neutralizing anti-apoptotic Bcl-2 family members. Rather than facilitating antigen escape from the phagosome, our strategy directly modulates the host cell death machinery to induce apoptosis, which may in turn promote antigen cross-presentation through apoptotic body uptake by dendritic cells. Collectively, these results indicate that a *mycobacterial* vector can effectively deliver a functional pro-apoptotic protein to macrophages. This finding provides a technical proof-of-principle rather than a complete vaccine evaluation and establishes a foundation for future engineering of BCG to deliver pro-apoptotic signals, although extensive immunological evaluation remains necessary.

Finally, we acknowledge that the present study is limited to in vitro observations in a single macrophage cell line and does not address the immunological outcomes, such as antigen presentation, T-cell priming, or protective efficacy, that would be required for a full vaccine evaluation [33,34,35]. Likewise, while the Sec pathway through which ASP directs protein secretion is the same in both *M. smegmatis* and BCG, and *M. smegmatis* is widely used as a surrogate for initial construct testing, it does not fully recapitulate BCG biology as a vaccine vector. Therefore, the ultimate validation of this strategy—including its immunogenicity and protective efficacy—must be performed in BCG-based constructs. Nevertheless, within these defined boundaries, our results clearly demonstrate that a *mycobacterial* vector can deliver a functional pro-apoptotic protein to macrophages, providing a technical validation for engineering BCG to modulate host cell apoptosis. These findings provide a clear experimental foundation and a specific construct for future BCG-based vaccine studies. Beyond this translational potential, this work makes two contributions. First, it demonstrates that BIK can be functionally secreted from a *mycobacterial* vector while retaining its pro-apoptotic activity, a finding not previously reported for this BH3-only protein. Second, it establishes an experimental framework using *M. smegmatis* as a surrogate host that can accelerate the initial screening of pro-apoptotic candidates before proceeding to BCG-based validation.

## Data Availability

The data presented in this study are contained within the article.
